# Publisher Correction: Selective enhancement of optical nonlinearity in two-dimensional organic-inorganic lead iodide perovskites

**DOI:** 10.1038/s41467-017-01766-z

**Published:** 2017-11-23

**Authors:** F. O. Saouma, C. C. Stoumpos, J. Wong, M. G. Kanatzidis, J. I. Jang

**Affiliations:** 1 Department of Physics, Applied Physics and Astronomy, State University of New York (SUNY) at Binghamton, PO Box 6000, Binghamton, NY 13902 USA; 2 Department of Chemistry, Northwestern University, 2145 Sheridan Road, Evanston, IL 60208 USA; 3 Department of Physics, Sogang University, Seoul, 04107 South Korea

*Nature Communications* **8**:742 doi:10.1038/s41467-017-00788-x; Article published online: 29 Sep 2017

In the PDF version of this article, Eq. 5 is missing all elements after the equals sign. The correct version of Eq. 5 is given below. The HTML version of the paper was correct from the time of publication.

$$\begin{array}{ccccc}\\ G_2\left( x \right) = & \frac{1}{{(2x)^6}}\left\{ \begin{array}{l}4[1 - (1 - x)^{3/2} - (1 + x)^{3/2}] - \frac{3}{4}x^2[(1 - x)^{ - 1/2} + (1 + x)^{ - 1/2}]\\ + 6x\{ (1 - x)^{1/2} - (1 + x)^{1/2}\} + 2[H(1 - 2x)(1 - 2x)^{3/2} + (1 + 2x)^{3/2}]\end{array} \right\}\\ \\ & + \frac{1}{{(2^{10}x^5)}}\left\{ {70x^2 + 3x[(1 - x)^{ - 1/2} - (1 + x)^{ - 1/2}] - \frac{1}{2}x^2[(1 - x)^{ - 3/2} + (1 + x)^{ - 3/2}]} \right\}\\ \end{array}$$

