# Supplementary material for: Selective enhancement of optical nonlinearity in two-dimensional organic-inorganic lead iodide perovskites
Source: Nat Commun. 2017 Sep 29;8:742. doi: 10.1038/s41467-017-00788-x (PMC5622136; doi:10.1038/s41467-017-00788-x)
Supplement: Supplementary file 1 — Supplementary Information [file 41467_2017_788_MOESM1_ESM.pdf]

Description of Supplementary Files

File Name: Supplementary Information
Description: Supplementary Notes, Supplementary Figures and Supplementary References

File Name: Peer Review File

Supplementary Note 1: Bandgap estimation of $(BA)_2(MA)_{n-1}Pb_nI_{3n+1}$ ($n = 1$–∞)

In our previous study[1], bandgaps of the title perovskites were roughly determined by extrapolating their absorption edges to the energy axis, yielding 1.50 eV ($n = \infty$), 1.91 eV ($n = 4$), 2.03 eV ($n = 3$), 2.17 eV ($n = 2$), and 2.43 eV ($n = 1$), respectively, at room temperature. However, the presence of excitonic absorption slightly below the fundamental band edge caused some uncertainty in the bandgap estimation of each compound. This effect could be less clear for the bulk perovskite ($n = \infty$) because the exciton line is not well resolved from the band edge; while the stability of excitons at room temperature is still under debate[2-4], the optical response of $MAPbI_3$ is excitonic. To evaluate the accurate dependence of $\chi^{(3)}$ on the bandgap, E_g, in this work we estimated E_g of our perovskites by eliminating the excitonic contribution that can be modelled with a Gaussian peak. The spectral location of the exciton peak was determined to match with the low-energy onset of the absorption edge while consistently keeping the width of the exciton peak ~52 meV for all the 2D perovskites and ~43 meV for $MAPbI_3$. These peaks are shown in Supplementary Figs. 1a–1e as black curves for $n = \infty$, $n = 4$, $n = 3$, $n = 2$, and $n = 1$, respectively, overlaid with the experimental absorption spectra (coloured traces).

Since the perovskites are direct-gap semiconductors[1], we plot $\alpha(E)^2E^2$ as a function of photon energy, E, in Supplementary Fig. 2a where $\alpha(E)$ is obtained by subtracting the exciton peak from the experimental absorption data of Supplementary Figs. 1a–1e. Note that the exciton peak is therefore absent, allowing us to unambiguously determine E_g, which are 1.60 eV ($n = \infty$), 1.89 eV ($n = 4$), 2.00 eV ($n = 3$), 2.14 eV ($n = 2$), and 2.40 eV ($n = 1$), respectively. For comparison, we also plot $\alpha(E)^2E^2$ for the bulk perovskite without spectral filtering (dashed trace), which indeed better reveals the presence of the exciton peak; therefore, we believe that it is reasonable to

assume a finite exciton binding energy for n = ∞. The exciton binding energy, E_{ex}, for each perovskite was also determined (Supplementary Fig. 2b). We note that the measured E_{ex} = 40±5 meV for n = ∞ is indeed reasonable consistent with not only the measured values by optical absorption[4] but also a precision measurement based on magnetoabsorption[5]. Both spectroscopic methods are not directly affected by the choice of the dielectric constant between ε_0 and ε_∞. Most of all, we confirmed that this issue of exciton binding energy[4] minimally affects the bandgap dependence of the third-order nonlinearity of the bulk perovskite (n = ∞); both n_2 vs. E_{g} and β vs. E_{g} plots for n = ∞ in the main text are well explained by the two-band model even with E_{ex} = 0. As expected, E_{ex} increases as the thickness of the perovskite layer reduces due to enhanced quantum confinement. As discussed in the main text and below, this exciton level can be directly excited by resonant two-photon absorption (2PA) in the 2D perovskites (n = 1–4). This resonant 2PA cannot be explained by a conventional two-band model if the band dispersion parameter, $x = E/E_{\text{g}}$, is defined using the fundamental bandgap, E_{g}: Excitonic 2PA occurs for x slightly below 0.5 due to a finite exciton binding energy, but x is only defined for $0.5 < x < 1$ for typical band-to-band transition[6].

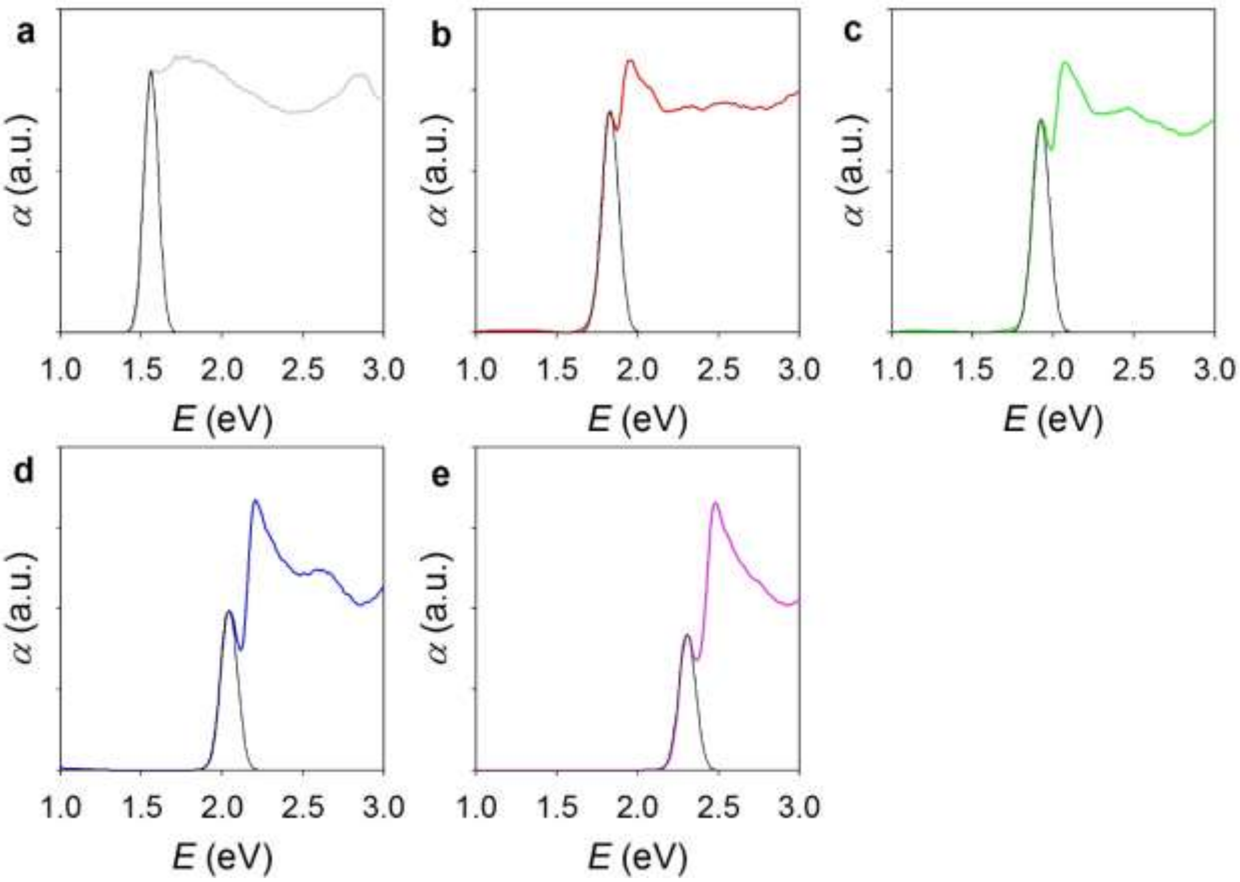

Supplementary Figure 1 | Experimental absorption spectra of perovskites. a-e, Absorption spectra for (a) $n = \infty$ (grey), (b) $n = 4$ (red), (c) $n = 3$ (green), (d) $n = 2$ (blue), and (e) $n = 1$ (purple), respectively, at room temperature. The black curves are exciton peaks modelled with a Gaussian shape.

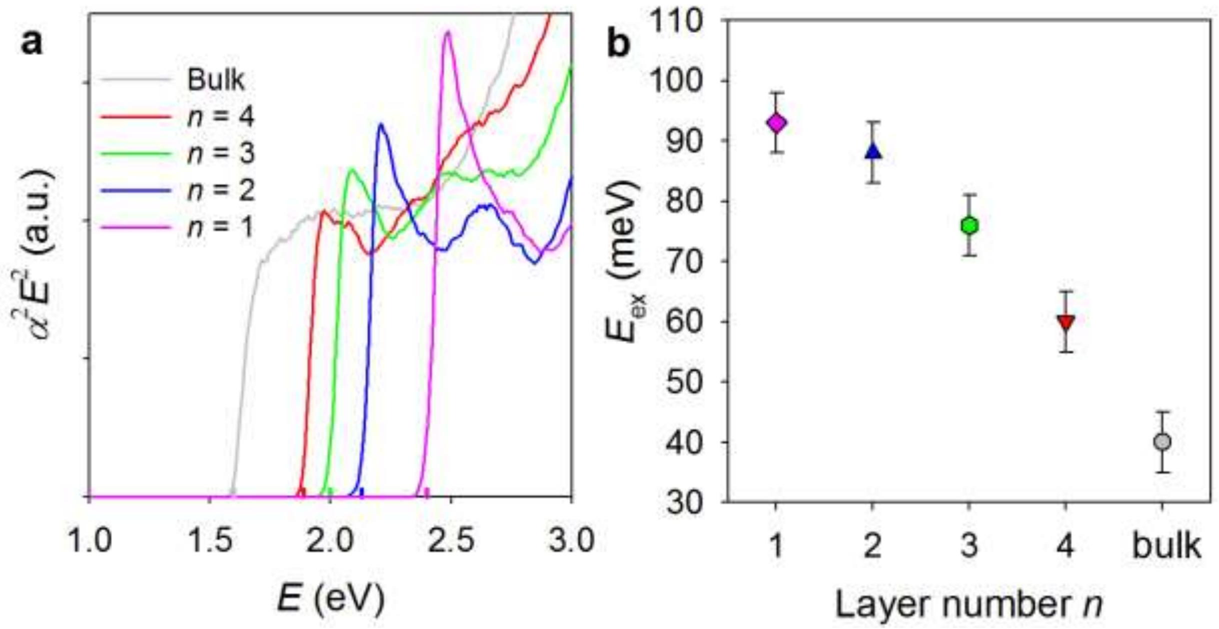

Supplementary Figure 2 | Band-edge absorption behaviours and exciton binding energies of perovskites. **a**, $\alpha(E)^2E^2$ vs. E to determine bandgaps by Tauc's fits after subtracting the excitonic contribution. The locations for the band edges are marked in the energy axis. The dashed trace corresponds to the bulk without spectral subtraction of the exciton peak. The presence of excitons is evident when compared with the grey trace. **b**, Exciton binding energy (E_{ex}) as a function of perovskite layer number at room temperature. The error bars in E_{ex} arise from uncertainty in Tauc's fits.

Supplementary Note 2: Crystal symmetry of $(BA)_2(MA)_{n\text{-}1}Pb_nI_{3n+1}$ (n = 1–4)

If crystallographic restrictions that demand an inversion centre are set aside, we can see that the layers with an even number of n are polar when viewed individually. This is illustrated by the shape of perovskite cavities which undergo a combination of in-phase and out-of-phase distortion. These distortions force the cavities to adopt either a distorted rhombus or regular square

shape when viewed along the crystallographic a axis (Fig. 1b in the main text). This representation is a good intuitive measure of the polarity in the 2D perovskites and indicates that polarization is cancelled out along the ac plane (equal number of squares and rhombi). However, along the b axis, polarization is nonzero (different number of squares and rhombi). Thus, overall polarization can be cancelled only if each layer completely cancels with its neighboring layer that exactly coincides with the crystallographic description; the layers are related through glide planes. This picture suggests that even the presence of minor defects, such as the ones listed below, may break the inversion symmetry, leading to a local polarization.

In fact, we have previously reported that the present set of compounds can crystallize in the corresponding polar space groups[1] although a margin of ambiguity of this assignment still remains. Structural defects arising from the flexible nature of the nanoscopically thin perovskite layers can thermally activate dynamic disorder of the perovskite lattice and most importantly twinning, a hallmark property of the perovskite crystal structure, which may easily smear out any long-range polarization effect. In light of the results presented here, however, we will follow the centrosymmetric assignment since it better justifies the experimental data as confirmed by very weak second harmonic radiation, i.e., $\chi^{(2)} = 0$. The notation in the main text is given in non-standard crystallographic space groups so that the lattice parameters follow the $c < a < b$ sequence for all the compounds. The polar vs. nonpolar assignment of the crystal structure of the halide perovskites, in general, is still under serious debate and the assignment given in the main text is not necessarily a conclusive one.

Supplementary Note 3: Kurtz-Perry powder method

During the discovery stage of novel nonlinear optical (NLO) materials, it is often challenging to make NLO measurements on single crystals, since suitably-sized crystals may not be readily available (issues of synthesis, cost, etc.). The Kurtz-Perry powder technique has become the most common method for estimating the optical nonlinearity of powder samples since its inception in the original work[7] of 1968. The major advantage of this technique is that it allows one to measure the absolute value of nonlinearity of a material before spending large amounts of time, effort, and resources to produce large single crystals; here, an effective value is obtained averaged over randomly distributed powders.

Although it is more popular in determining the second-order NLO coefficient, the technique can be readily generalized to evaluate third-order NLO effects[8]. It is generally accepted that the powder technique yields a fairly reliable assessment of a specimen if the measurement is made with respect to a well-established reference powder for which the NLO coefficients are known. In our experiments, a benchmark NLO material of optical-quality $AgGaSe_2$ powder was used. Within the measurement scheme, scattering by powder particles is unavoidable unless the specimen is immersed in an index-matching fluid, which is not always available. This scattering effect can be properly corrected based on a theoretical model[9] or minimized by conducting experiments under our carefully designed condition.

Our experiments were performed in the ambient condition on densely packed powders of size, $d = 90$–125 μm, which is much larger than the optical wavelengths associated with input as well as third harmonic generation (THG) and/or photoluminescence (PL) from the sample. In this case, Mie scattering is dominant and the maximum scattering direction is highly oriented along

the input-beam direction[9]. In order to minimize this scattering effect, we therefore employed reflection geometry in which the mean free path for backward scattering is comparable to the powder particle size[9]; this essentially corresponds to a single scattering event of the incident beam by only one layer of powders and multiple scattering is only significant along the forward direction. Since our size of a capillary tube containing powders is also much larger than the particle size, the PL and THG are only generated from the front surface of the tube, which can be effectively captured under our reflection geometry. This condition ensures minimal effects by scattering on the estimation of NLO coefficients of our samples. As a clear evidence, we found that the measured 2PA coefficient, β, of the reference powder is consistent with that determined from a bulk single crystal within a factor of two as described in the main text. We also confirmed that the measured optical signals from the sample powder are fairly consistent when the illumination spot was scanned over the various portions of the capillary tube.

We emphasis that our powder method yields more reliable NLO coefficients, compared with a typical *Z*-scan method using polycrystalline thin films. While thin-film measurement may reduce minor scattering effects, it can lead to an inaccurate estimation of optical nonlinearity because of submicron-size grains typically present in perovskite films. It is well known that such fine grains in a thin film causes significant enhancement in the nonlinear optical response due to extra charges accumulated on the grain boundaries[10]. For example, recent experiments using thin perovskite films also yielded very large optical nonlinearity[11–13], which is far beyond the theoretical prediction of the two-band model[6]. Although such a large effect is desirable for actual NLO applications, it is clearly not the intrinsic property of the perovskites.

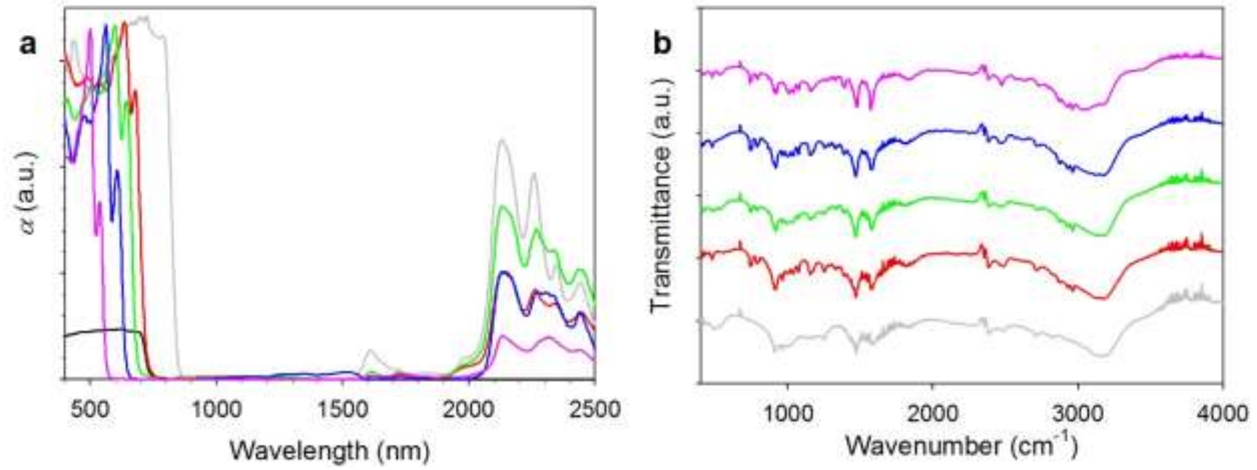

Supplementary Figure 3 | Linear absorption spectra and Fourier transform infrared spectra . **a**, Absorption spectra obtained from diffuse reflectance measurements converted using Kubelka-Munk function ($\alpha/S = (1-R)^2/2R$) for $AgGaSe_2$ (black), $n = \infty$ (grey), $n = 4$ (red), $n = 3$ (green), $n = 2$ (blue), and $n = 1$ (purple), respectively. The chalcogenide has a wide transparency range from the fundamental bandgap extending to the IR up to its vibrational mode, whereas the organic-inorganic hybrid perovskites have the typical absorption band in the range of 2000 nm–2500 nm due to activation of organic cations. This IR absorption effect indeed causes fundamental loss in the perovskites, thereby causing relatively inefficient THG as evidenced by Supplementary Fig. 4. Nevertheless, our 2D perovskites outshine $AgGaSe_2$ over the entire monitoring range (Fig. 2a in the main text). **b**, Fourier transform infrared (FTIR) spectra of the perovskites in the range of 2500 nm–25000 nm (400 cm^{-1}–4000 cm^{-1}), showing the molecular vibration modes of the BA and MA cations. Each FTIR spectrum is vertically translated for clarity. Being ubiquitous to the hybrid halide perovskites, these are not extrinsic subgab states. Most importantly, we emphasize that our characterization of nonlinear refractive indices based on THG at 2700 nm is barely affected by this minor effect; the corresponding IR transmittance is higher than 90%. The minimum transmittance of 30 %, common to all the perovskites, occurs around 3200 cm^{-1}.

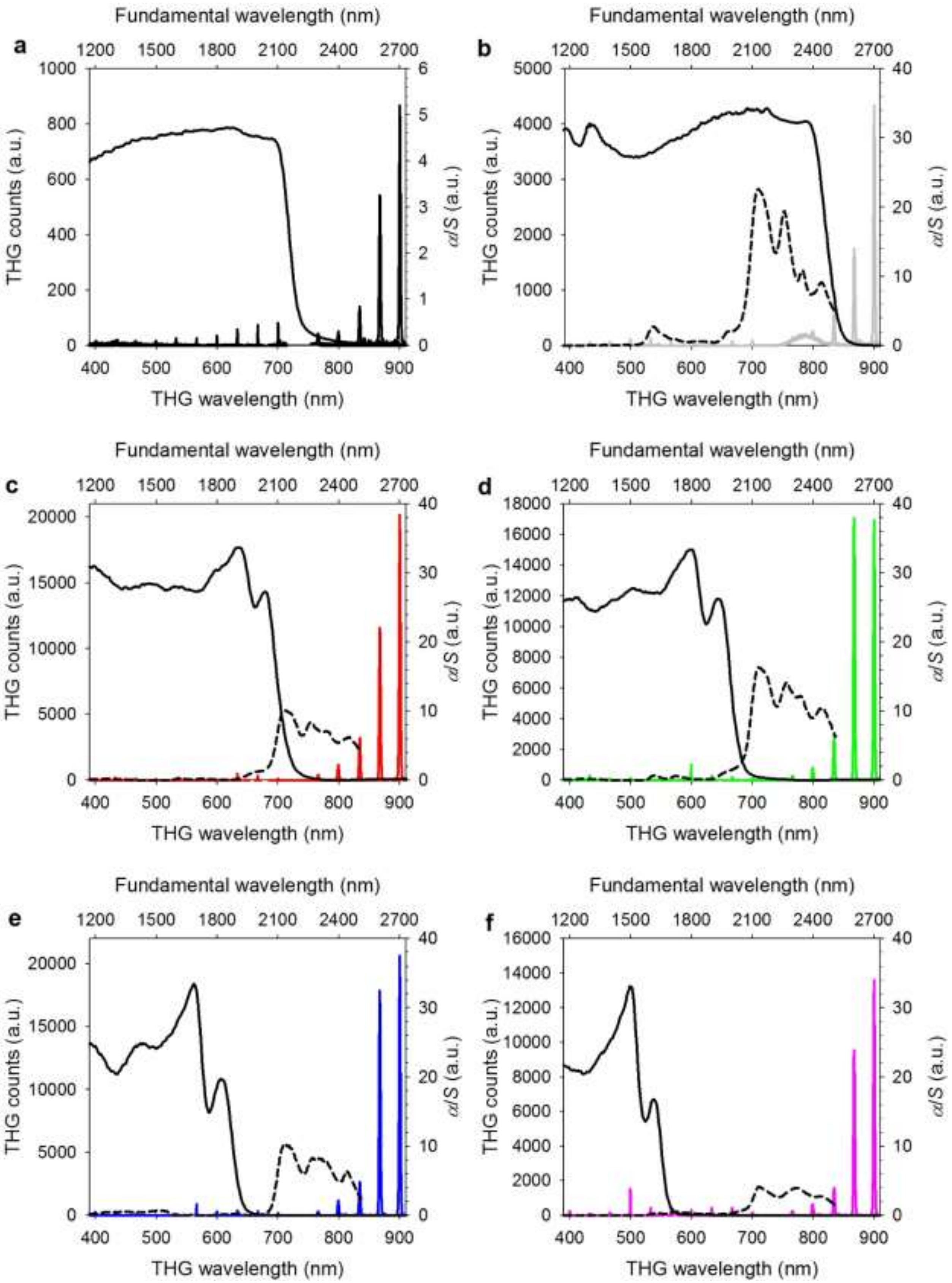
a
b
c
d
e
f
Fundamental wavelength (nm)
THG wavelength (nm)
THG counts (a.u.)
α/S (a.u.)

Supplementary Figure 4 | Wavelength-dependent THG. **a-f**, Data for (a) $AgGaSe_2$ (black), (b) *n* = ∞ (grey), (c) *n* = 4 (red), (d) *n* = 3 (green), (e) *n* = 2 (blue), and (f) *n* = 1 (purple), respectively, when the input wavelength is varied from $\lambda =$1200 nm to 2700 nm. The solid and dashed traces in each panel are linear absorption spectra arising from bandgap absorption and organic-cation excitation, respectively. The THG counts plummet when the corresponding THG wavelength approaches to the fundamental bandgap because of significant reabsorption of THG by the sample itself. The THG efficiency of our perovskites is also affected by fundamental absorption at the IR (dashed trace) that in turn reduces the overall THG counts at the corresponding THG wavelength, $\lambda_{\mathrm{THG}} = \lambda/3 =$ 700 nm – 833 nm; note that the THG counts are relatively smaller in this range. Our perovskites are therefore most efficient in the mid-IR.

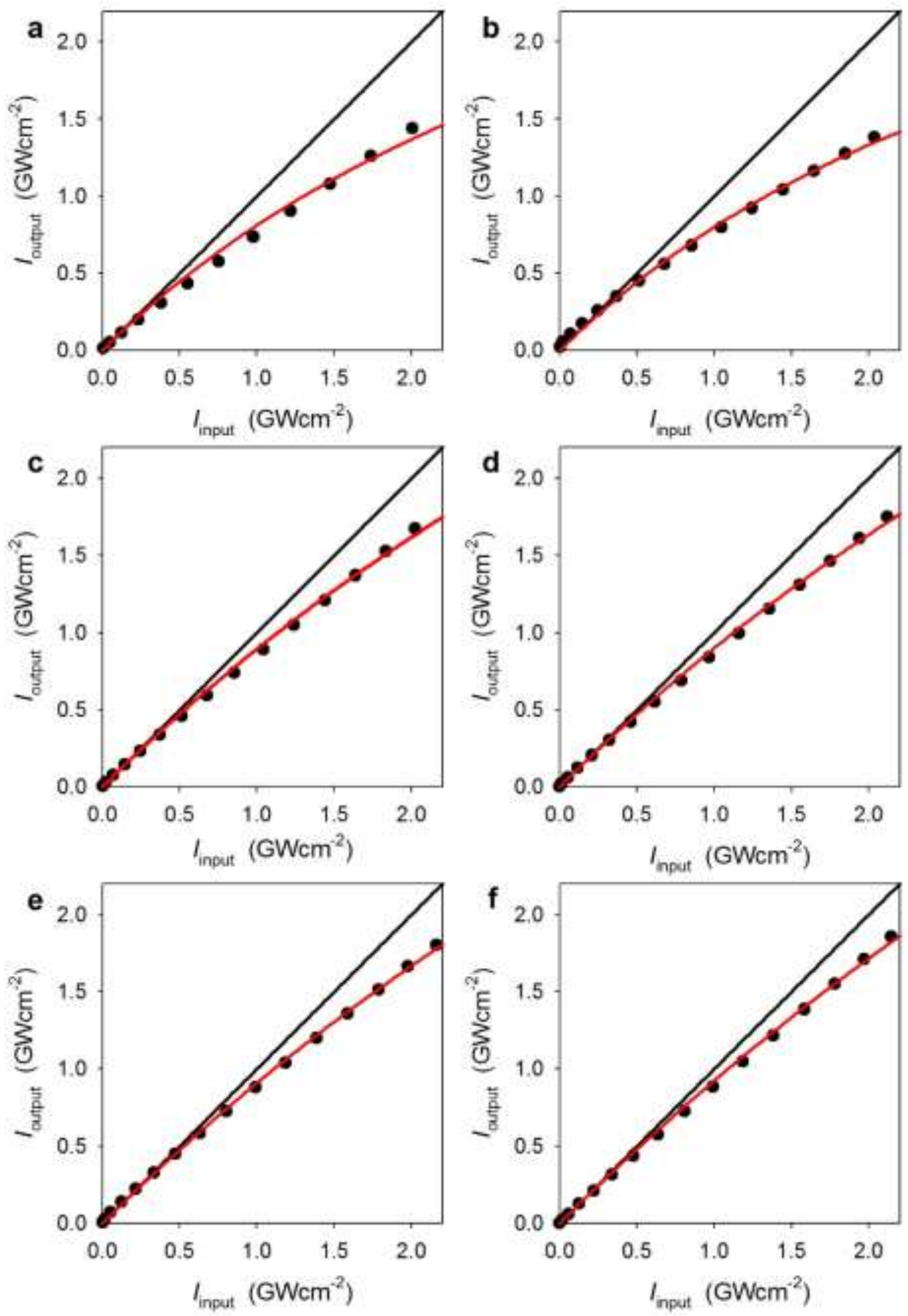

Supplementary Figure 5 | 2PA in perovskites. **a-f**, I_{input} vs. I_{output} (dots) for (a) $AgGaSe_2$, (b) $n = \infty$, (c) $n = 4$, (d) $n = 3$, (e) $n = 2$, and (f) $n = 1$ at $\lambda = 1064$ nm, superimposed by 2PA fits (red curves), yielding $\beta = 39.9$ cmGW^{-1}, 46.5 cmGW^{-1}, 21.9 cmGW^{-1}, 20.7 cmGW^{-1}, 18.4 cmGW^{-1}, and 15.3 cmGW^{-1}, respectively. The black line corresponds to no 2PA. Smaller deviation from the black line indicates a higher LIDT.

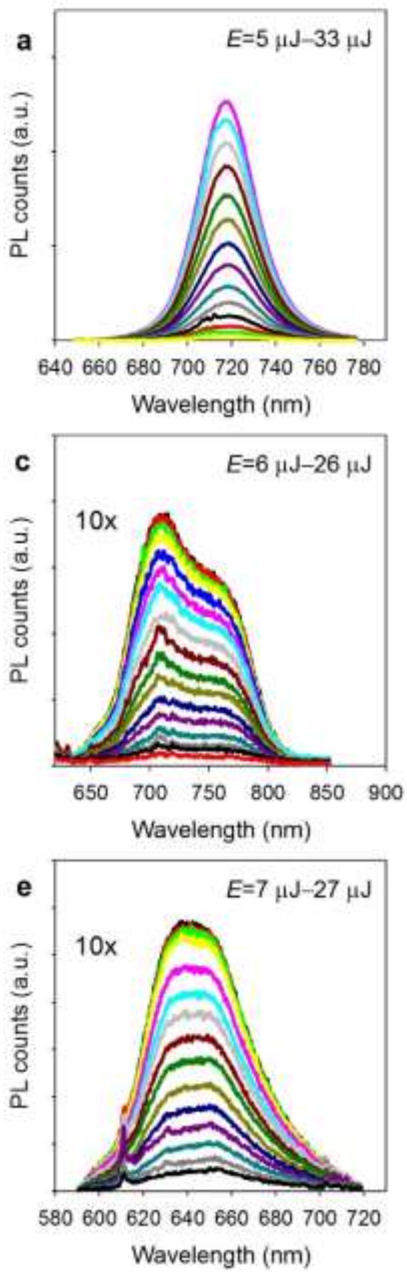

Supplementary Figure 6 | 2PA-induced PL spectra at $\lambda = 1064$ nm under various excitation energies, *E*. **a-f**, Data specified for (a) $AgGaSe_2$, (b) $n = \infty$, (c) $n = 4$, (d) $n = 3$, (e) $n = 2$, and (f) $n = 1$, respectively. The $n = 1$ perovskite yields much weaker PL response when cold excitons are resonantly generated by 2PA. Minor peaks around 610 nm and 710 nm are from background radiation, each from a computer monitor and second-order diffraction of the frequency-tripled Nd:YAG radiation, respectively.

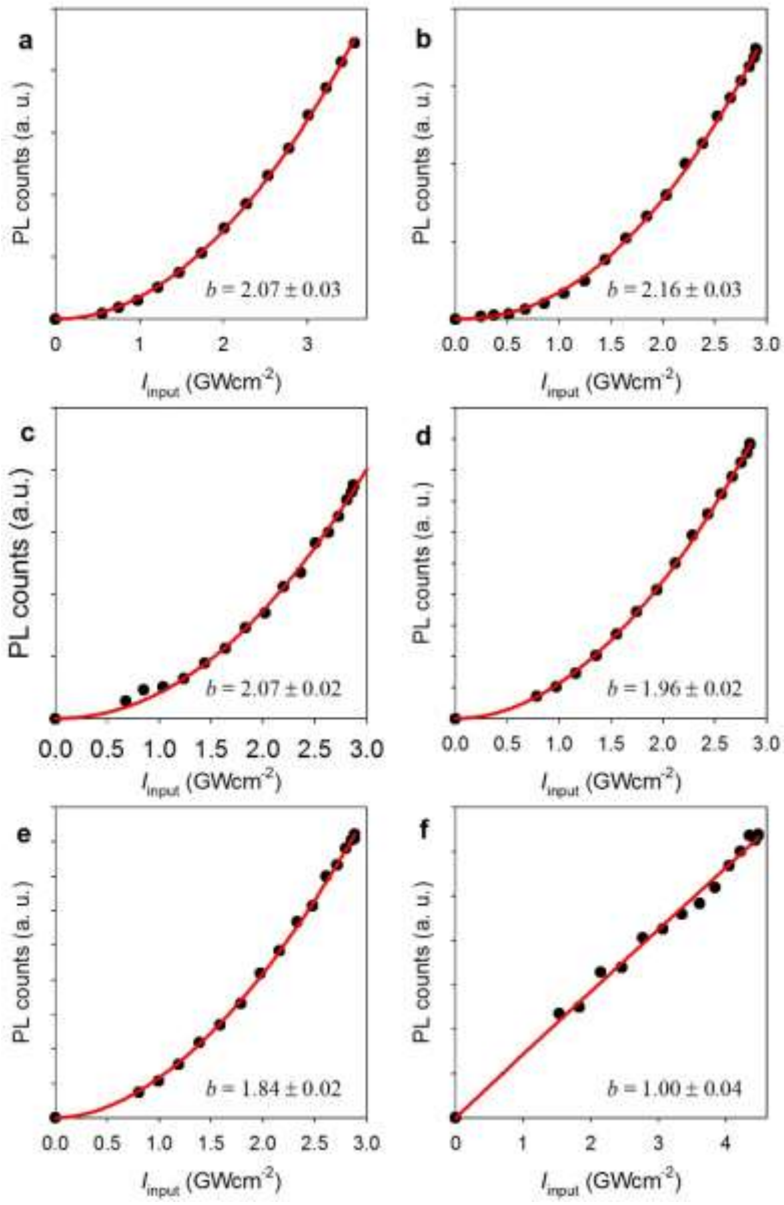

Supplementary Figure 7 | Intensity-dependent PL under 2PA. a-f, Data (dots) for (a) $AgGaSe_2$, (b) $n = \infty$, (c) $n = 4$, (d) $n = 3$, (e) $n = 2$, and (f) $n = 1$, respectively. The red traces are power-law fits, $y = ax^b$, where each critical exponent is shown with uncertainty arising from the standard deviation of each fit. Note that $n = 1$ exhibits a linear dependence ($b = 1.00$). This trend indicates that the PL is a free-to-bound type[14] when cold excitonic matter is directly generated by resonant 2PA. A slight deviation of the critical exponent of $n = 2$ from $b = 2$ may also indicate that this type of recombination is partially involved due to the proximity effect.

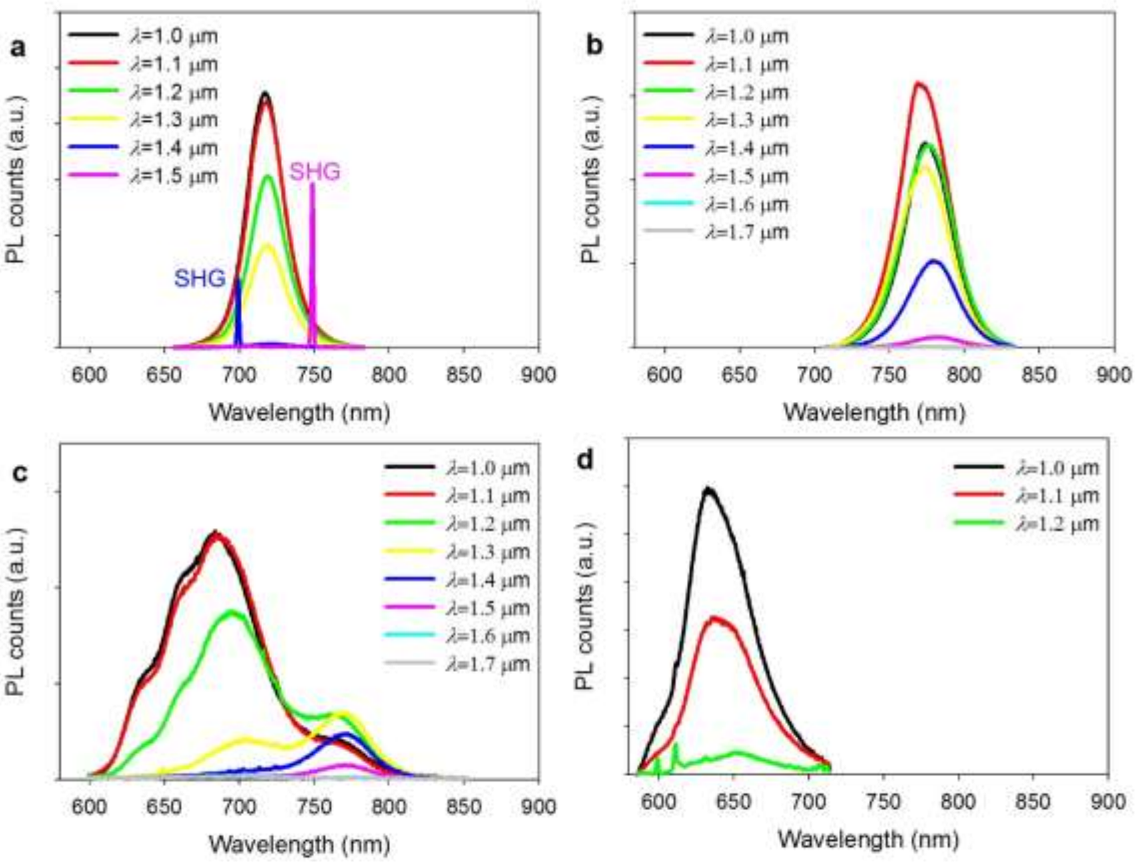

Supplementary Figure 8 | 2PA-induced PL under various excitation wavelengths, λ. a-d, Data from (a) $AgGaSe_2$, (b) $n = \infty$, (c) $n = 3$, and (d) $n = 2$, respectively. Bandgap engineering is apparent from the spectral shift of the PL in the Ruddlesden-Popper series. The additional low-energy shoulder around 780 nm in (c) is observed from $n = 3$ and $n = 4$ compounds only as more detailed in Supplementary Fig. 10. Unlike the perovskites, $AgGaSe_2$ is noncentrosymmetric, and therefore, exhibits sharp SHG at λ_{SHG} = 700 nm and λ_{SHG} = 750 nm in response to fundamental radiation at λ = 1400 nm and λ = 1500 nm, respectively. The PL spectra from the thinnest perovskite ($n = 1$) at λ = 1000 nm and 1100 nm are similar to those shown in Supplementary Fig. 6f. The 2PA dispersion of $n = 1$ was not accessible because of the experimental limitation, i.e., only one data point of λ = 1000 nm being above the bandgap of the compound, E_g = 2.40 eV (~516 nm). Note that Supplementary Fig. 6f shows the PL under resonant 2PA at the exciton level of the compound, which is ~93±5 meV below the bandgap.

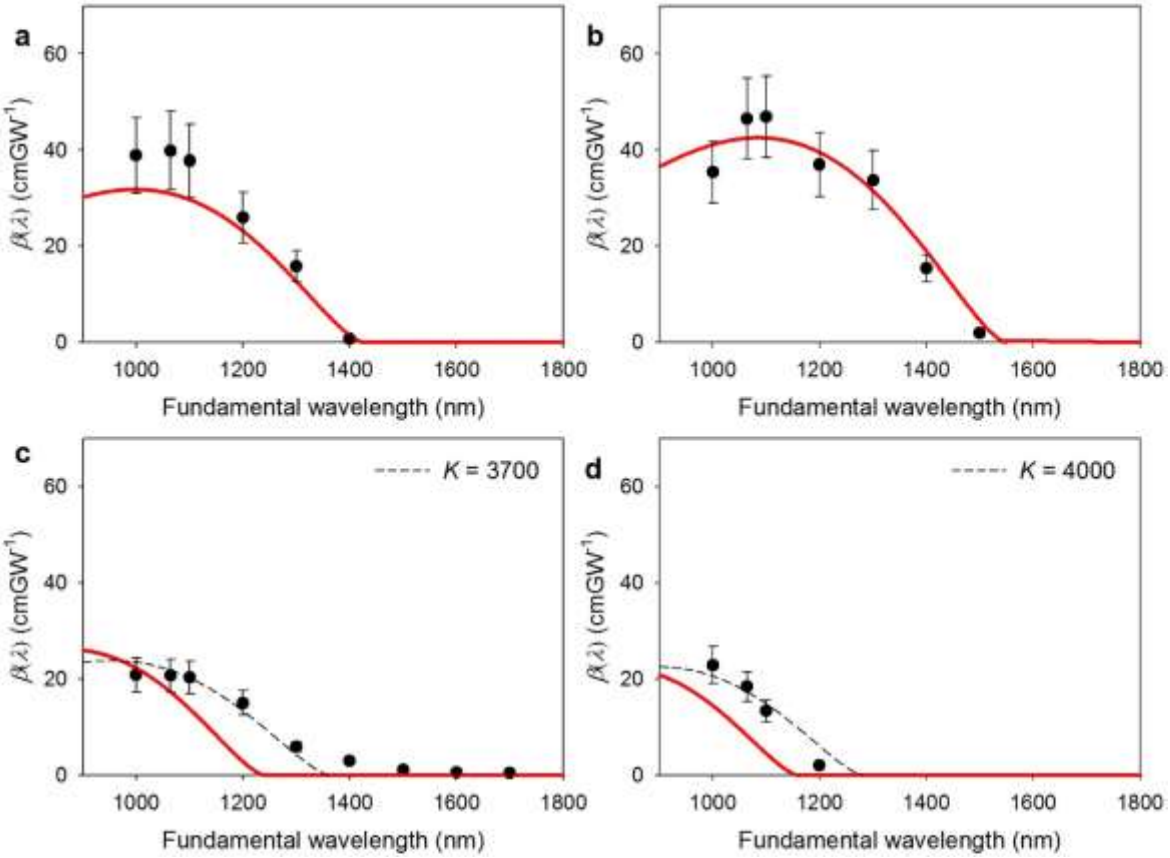

Supplementary Figure 9 | $\beta(\lambda)$ determined by the PL method. a-d, Data (dots) for (a) $AgGaSe_2$, (b) $n = \infty$, (c) $n = 3$, and (d) $n = 2$, respectively. The red curves correspond to the two-band model with K = 5000 from the main text. Note that experimental 2PA dispersions of our compounds and the reference chalcogenide can be consistently described by the single Kane parameter within the error-bar range arising from uncertainty in the powder size, except for $n = 2$ as shown in part (d). As discussed in the main text, 2PA in $n = 2$ is rather enhanced by the spectral proximity to the exciton line. The dashed curves are the two-band model in terms of optical gap determined from the onset of excitonic transition in the 2D perovskites (Supplementary Fig. 1). K is also slightly adjusted to fit the overall wavelength dependence. We found that the experimental 2PA dispersion is better explained when using the optical gap for the 2D perovskites with K = 3500–4000 – see also Fig. 4c in the main text. 2PA of defect-induced transition for n = 3 and n = 4 is evident even with $\lambda > 1400$ nm up to 1700 nm.

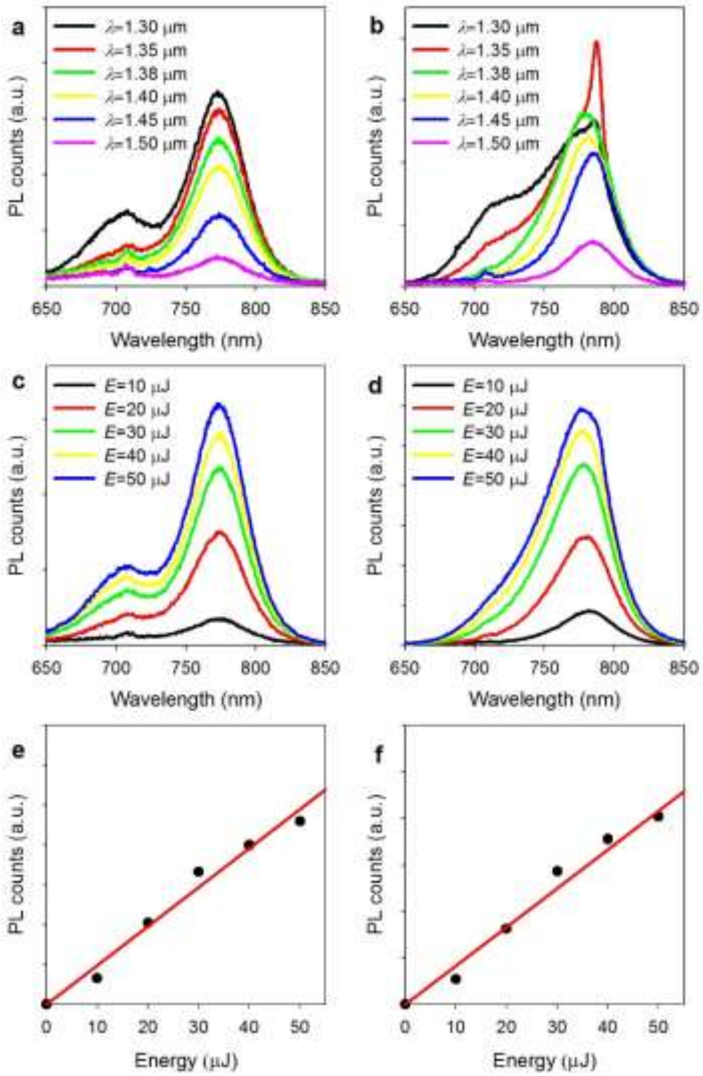

Supplementary Figure 10 | PL by defect-induced 2PA. a,b PL spectra when the excitation wavelength is tuned below the exciton line for (a) n = 3 and (b) n = 4. Amplified emission can be seen for n = 4 when the input photon energy is two-photon resonant with the exciton level. Note that spectral locations for the defect-induced PL in these two perovskites are similar (~780 nm). This indicates the same nature of defects which may arise from some organic molecules escaping, leaving behind a defective layer. **c,d**, Intensity-dependent PL spectra from (c) n = 3 at $\lambda = 1350$ nm and (d) n = 4 at λ = 1380 nm under several excitation levels, where the defect-induced PL is clearly observed. The corresponding power dependence is shown in **e** and **f**. A linear response (red) again indicates that the nature of transition is free-to-bound type[14].

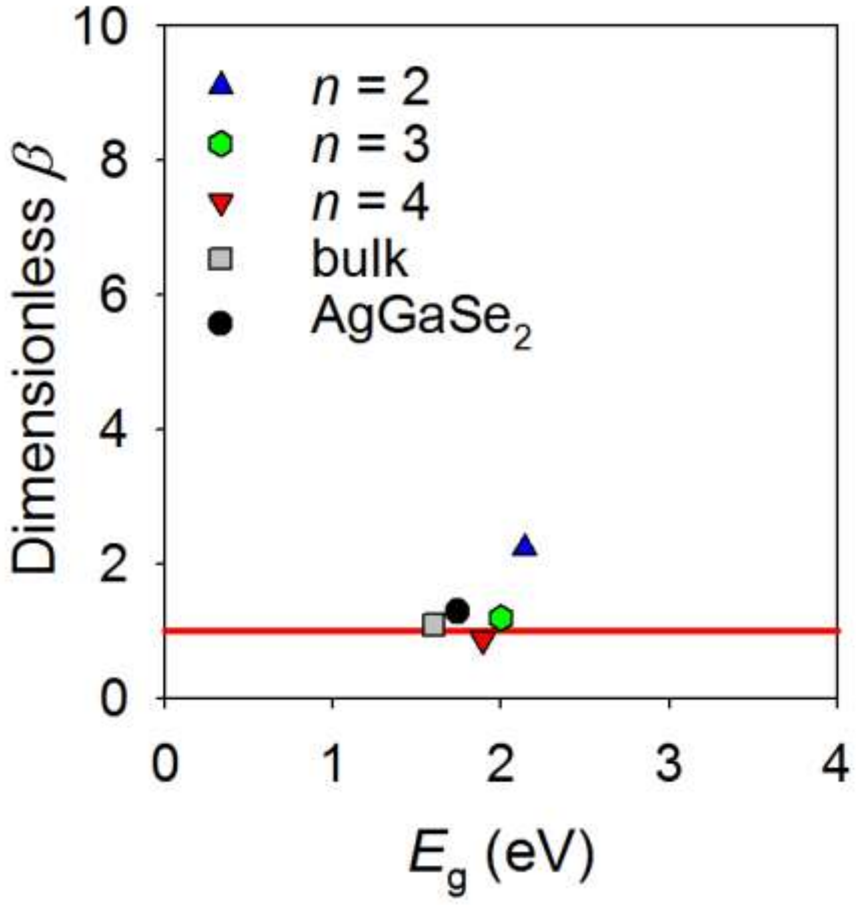

Supplementary Figure 11 | Impact of quantum confinement on β of the 2D perovskites. The effect of dimensional reduction is best demonstrated by dimensionless β, which can be obtained by multiplying the scaled β in the main text (Fig. 3d) by E_g^3. Regardless of E_g, dimensionless β is simply normalised to unity (red line), which is the two-band model[6]. The data point for *n* = 1 is missing because in this case 2PA is purely excitonic and $F_2(x)$ is not well defined. A slight enhancement for *n* = 2 can be attributed to the spectral proximity to the exciton line (see Supplementary Fig. 9d). Unlike n_2 (Fig. 2d in the main text), the 2PA efficiency is not significantly affected by quantum confinement.

Supplementary References

1. Stoumpos, C. C. *et al*. Ruddlesden-Popper hybrid Lead Iodide perovskite 2D homologous semiconductors. *Chem. Mater*. **28**, 2852–2867 (2016).

2. Savenije, T. J. *et al.* Thermally activated exciton dissociation and recombination control the carrier dynamics in organometal halide perovskite. *J. Phys. Chem. Lett.* **5**, 2189–2194 (2014).

3. Miyata, A. *et al.* Direct measurement of the exciton binding energy and effective masses for charge carriers in organic-inorganic tri-halide perovskites. *Nature Phys.* **11**, 582–587 (2015).

4. Manser, J. S., Christians, J. A. & Kamat, P. V. Intriguing optoelectronic properties of metal halide perovskites. *Chem. Rev.* **116**, 12956–13008 (2016).

5. Hirasawa, M., Ishihara, T., Goto, T., Uchida, K. & Miura, N. Magnetoabsorption of the lowest exciton in perovskite-type compound $(CH_3NH_3)PbI_3$. *Phys. B: Cond Matter* **201**, 427–430 (1994).

6. Sheik-Bahae, M., Hutchings, D. C., Hagan, D. J. & van Stryland, E. W. Dispersion of bound electronic nonlinear refraction in solids. *IEEE J. Quantum Electron.* **27**, 1296–1309 (1991).

7. Kurtz, S. K. & Perry, T. T. A powder technique for the evaluation of nonlinear optical materials. *J. Appl. Phys.* ***39***, 3798–3813 (1968).

8. Haynes, A. S. *et al*. Phase transition, conformational exchange, and nonlinear optical third harmonic generation of ACsP$_2$Se$_8$ (A = K, Rb, Cs). *Chem. Mater*. **28**, 2374–2383 (2016).

9. Aramburu, I., Ortega, J., Folcia, C. L. & Etxebarria, J. Second harmonic generation by micropowders: a revision of the Kurtz-Perry method and its practical application. *Appl. Phys. B* **116**, 211–233 (2014).

10. Han, Y. B., Han, J. B., Ding, S., Chen, D. J. & Wang, Q. Q. Optical nonlinearity of ZnO microcrystallite enhanced by interfacial state. *Opt. Express* **13**, 9211–9216 (2005).

11. Kalanoor, B. S. *et al*. Third-order optical nonlinearities in organometallic methylammonium lead iodide perovskite thin films. *ACS Photon*. **3**, 361–370 (2016).

12. Zhang, R. *et al*. Nonlinear optical response of organic-inorganic halide perovskites. *ACS Photon*. **3**, 371–377 (2016).

13. Johnson, J. C., Li, Z., Ndione, P. F. & Zhu, K., Third-order nonlinear optical properties of methylammonium lead halide perovskite films. *J. Mater. Chem. C* **4**, 4847–4852 (2016).

14. Schmidt, T., Lischka, K. & Zulehner, W. Excitation-power dependence of the near-band-edge photoluminescence of semiconductors. *Phys. Rev. B* **45**, 8989–8994 (1992).
